# Low-magnitude high-frequency loading, by whole-body vibration, accelerates early implant osseointegration in ovariectomized rats

**DOI:** 10.3892/mmr.2014.2597

**Published:** 2014-09-23

**Authors:** YONG-QIANG LIANG, MENG-CHUN QI, JIANG XU, JUAN XU, HUA-WEI LIU, WEI DONG, JIN-YUAN LI, MIN HU

**Affiliations:** 1 Department of Stomatology, Chinese PLA General Hospital, Beijing 100853, P.R. China; 2 College of Stomatology, Hebei United University, Tangshan, Hebei 063000, P.R. China; 3 Department of Stomatology, Tongchuan City People’s Hospital, Tongchuan, Shaanxi 727100, P.R. China

**Keywords:** low-magnitude high-frequency loading, whole-body vibration, osteoporosis, dental implants, osseointegration, rat

## Abstract

Osteoporosis deteriorates jaw bone quality and may compromise early implant osseointegration and early implant loading. The influence of low-magnitude, high-frequency (LMHF) vibration on peri-implant bone healing and implant integration in osteoporotic bones remains poorly understood. LMHF loading via whole-body vibration (WBV) for 8 weeks has previously been demonstrated to significantly enhance bone-to-implant contact, peri-implant bone fraction and implant mechanical properties in osteoporotic rats. In the present study, LMHF loading by WBV was performed in osteoporotic rats, with a loading duration of 4 weeks during the early stages of bone healing. The results indicated that 4-week LMHF loading by WBV partly reversed the negative effects of osteoporosis and accelerated early peri-implant osseointegration in ovariectomized rats.

## Introduction

Osteoporosis, characterized by a reduction in bone mass, and deterioration of bone microarchitecture due to increased bone resorption, is a systemic condition that can lead to implant loosening and compromise the long-term success of dental implants (1,2). The use of dental implants in osteoporotic patients is controversial. A number of clinical studies have suggested that osteoporosis may not be an absolute contraindication for dental implants (3,4). However, growing evidence supports the negative effect of osteoporosis on implant osseointegration, and various investigations have indicated that peri-implant bone formation and implant integration are severely deteriorated under osteoporotic conditions (5–7). To overcome the negative effects of osteoporosis, numerous pharmaceutical agents, including estrogen (7), bisphosphonates (2,8–10), simvastatin (11) and parathyroid hormone (PTH) (12) have been applied, and promising results have been obtained. The aforementioned studies have encouraged further exploration of alternative methods to improve bone implant integration under osteoporotic conditions.

A non-pharmacological intervention, involving mechanical stimulation by low-magnitude high-frequency (LMHF) loading by whole-body vibration (WBV), has drawn much attention (13–18). Abundant evidence from clinical and preclinical studies supports the anabolic potential of LMHF loading on bone tissue. LMHF vibration has been demonstrated to improve bone mass and muscle strength in disabled children (13), accelerate bone healing (14) and enhance bone quality and bone strength in osteoporotic animals (15,16). Furthermore, LMHF vibration is also able to promote peri-implant bone formation and implant osseointegration in normal animals. In two studies by Ogawa *et al* (17,18), LMHF loading significantly increased bone-to-implant contact and peri-implant fraction in rats, and the effects of the vibration was crucially influenced by the loading time of each vibration and total loading duration. Despite these studies, the influences of LMHF vibration on peri-implant bone healing and implant integration in osteoporotic bones remains poorly understood.

Two studies by Akca *et al* (19) and Chen *et al* (20) provided clarification. These studies demonstrated that LMHF loading by WBV significantly increased peri-implant bone regeneration (19), bone-to-implant contact, maximum push-out force and interfacial shear strength (20) of the implants in ovariectomized rats. Although these studies provided valuable information, they did not cover the 4-week loading duration. As loading duration is a key influencing factor for vibration stimulation (18,21,22), further studies are required to elucidate the influence of varied durations, in order to get direct evidence on the anabolic effects of LMHF loading on implant osseointegration in osteoporotic bones.

The aim of the present study was to investigate LMHF loading by WBV with a 4-week loading duration, and to observe whether it is able to reverse the negative effects of osteoporosis and promote peri-implant bone healing and osseointegration in ovariectomized rats. If LMHF vibration with a short loading duration at the early stage of bone healing, is able to exert a similar anabolic bone response as it does in healthy animals, it may benefit early implant osseointegration and facilitate early implant loading.

## Materials and methods

### Animal grouping and ovariectomy

Thirty-six 12-week-old adult female Sprague Dawley rats with a mean body weight (BW) of 246.20±8.44 g were used in the current study (the Animal Center of Military Medical Science Academy of the PLA, Beijing, China). The BW of the animals was measured once per week throughout the experiment. All experimental procedures were approved by the Animal Care and Use Committee of Hebei United University, Tangshan, China (no. SCXK; Jing 2009-0004) and was in accordance with the guidelines for the Use of Laboratory Animals, as denoted by Zimmermann (23).

The animals were randomly divided into three groups (n=12 in each group): The sham, OVX and OVX-V groups. Following 1 week of acclimatization, the rats in the OVX and OVX-V groups received a bilateral ovariectomy, while the rats in the sham group underwent sham operations as per the methods of previous studies (2,7). Following surgery, the fascia and skin were sutured in layers and the animals were administrated with analgesia and prophylactic antibiotics at the time of surgery and for 3 days postoperatively.

### Implantation and vibration

**Figure 1:**
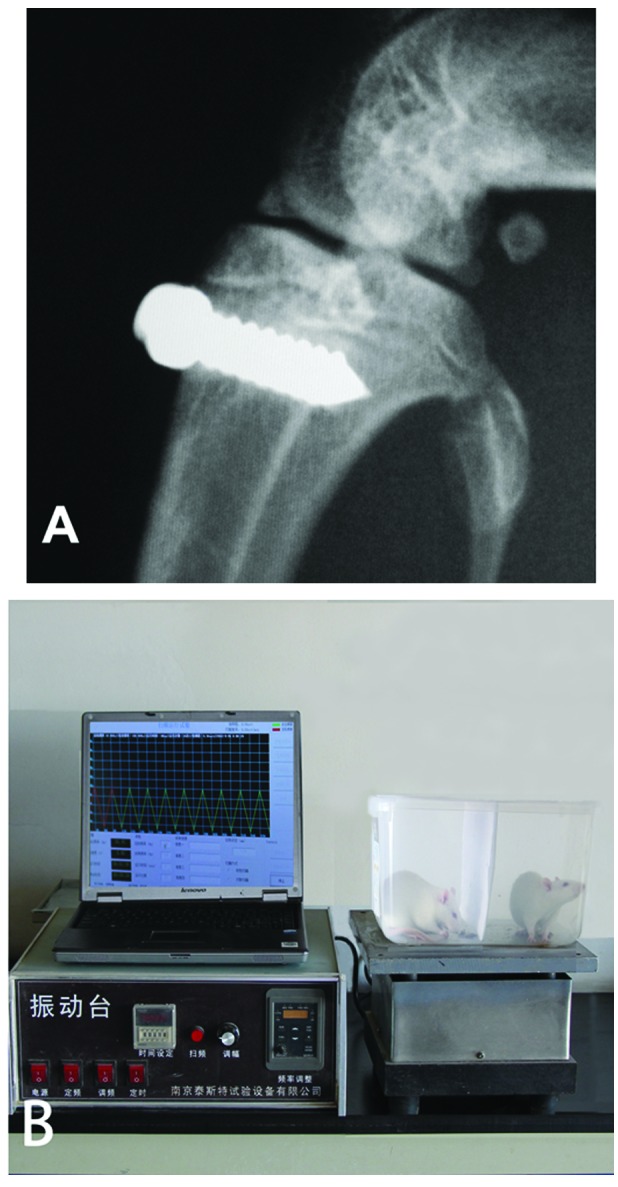
(A) X-ray demonstrating insertion of titanium implant in the proximal metaphysis of the tibia and (B) the vibrating device.

Four weeks after ovariectomy (20), all animals received a bilateral insertion of implants in the proximal metaphyses of their tibiae under general anesthesia. The implants used were custom-made screw-type titanium implants (Ø2×7 mm) processed from a titanium rod (99.8% Ti; National Engineering Research Center for Biomaterials, Sichuan University, Chengdu, China) and no surface modification was made. Briefly, incisions (10 mm in length) were made at the bilateral proximal metaphyses of the tibiae, and implantation holes (~1.9 mm in diameter) were drilled with a dental bur (Strong204, Seashin Precision Industrial Co., Taegu, South Korea) under constant saline irrigation. The implants were then screwed into the holes (Fig. 1A) and the soft tissues were sutured in layers. Analgesia and prophylactic antibiotics were also administrated to the animals, as described.

Mechanical intervention was delayed for one week to allow for primary bone healing (20). LMHF loading was applied to the OVX-V group by WBV, with a custom-made vibrating device (Testsky Ltd., Nanjing, China). The vibration regime was 45 hz, 0.2 g, as has been used in previous studies (15–18,20,24). Briefly, the cage containing the rats was fixed on the platform of the device and the rats were treated for 30 min/day, 7 days/week (Fig. 1B). The vibration treatment was performed for 4 weeks. Following treatment, the rats were sacrificed by cervial dislocation and the specimens were resected for examination. The animals in the sham and OVX groups received no vibration treatment, and were housed under the same conditions.

### Dual energy X-ray absorptiometry (DEXA)

An XR36 DEXA bone densitomiter (Norland XR36, Norland Medical Systems, Inc., Fort Atkinson, WI, USA) was used for the evaluation of the bone mineral density (BMD) at the start and end of experiments. The animals were placed in the scanner under general anesthesia and the femurs were scanned in a standard position with the posterior surface of the bone facing the scanner plate. The analysis of BMD was performed using a small subject program using a regional high resolution mode. The precision of the measurements of this DEXA technique was ±2.0%.

### Fluorescent labeling

To evaluate the bone dynamic mineralization, fluorescent labeling was performed using two fluorescent agents, tetracycline hydrochloride (yellow) and calcein (green; both from Sigma, Poole, Dorset, UK). Tetracycline hydrochloride (30 mg/kg) was administrated subcutaneously at 14 days prior to sacrifice once per day for 2 days, while calcein (6 mg/kg) was administrated at 4 days prior to sacrifice with the same administration protocol.

### Sample preparation

At the end of experiment, the tibiae and femurs of the rats were harvested. One tibia of each rat was processed for static and dynamic bone histomorphometric analyses, while the other was used for biomechanical testing. The specimens were fixed in 10% neutral buffered formalin for five days, dehydrated in increasing gradients of alcohol, and embedded in methylmethacrylate resin (Chengdu Kelong Chemical Co., Ltd., Chengdu, China). Undecalcified ground sections were obtained at a final thickness of 30 μm using an Exakt 300 sawing machine and grinding equipment (Exakt Advanced Technologies GmbH, Norderstedt, Germany). For evaluation of the osseointegration of implants in the tibiae, the sections were processed along the longitudinal direction of tibiae and the axis of the implant (2). The sections were initially examined under a laser scanning confocal microscope (LSCM; FV1000, Olympus Corporation, Tokyo, Japan) to evaluate the bone dynamic indices, and then they were stained with Toluidine blue (Sigma) and examined under a light microscope (Olympus Corporation) for evaluation of the bone static indices. All quantitative measurements were performed within a cancellous area of the tibiae.

### Bone static histomorphometry

**Figure 2:**
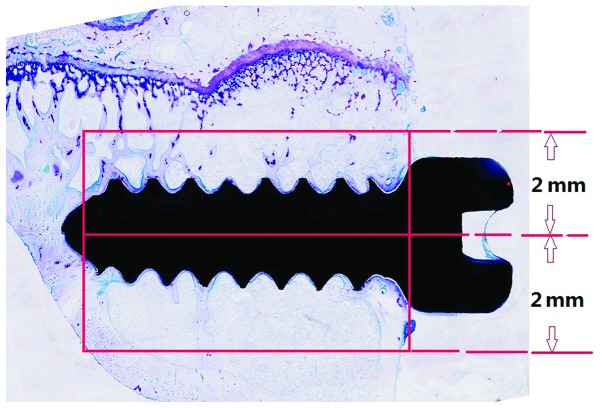
Region of interest for evaluation of BV/TV. The area is defined as a rectangular region within 2 mm from the axis of the implant.

Histomorphometric analysis was performed with a semi-automated digitizing image analyzer system, consisting of an Eclipse E600 stereoscopic microscope, a computer-coupled Digital Eclipse DXM 1200 digital camera and NIS-Elements F 2.20 imaging software (Nikon Corporation, Tokyo, Japan) (2). Bone static indices included the following: Bone-to-implant contact (BIC, %) = summation of the lengths of direct bone-to-implant contact interface/the length of total implant surface; peri-implant bone fraction (BF, %) = the percentage area of the bone within the rectangular region 2.0 mm from the axis of the implant; and thickness of the bone lamellae (TBL, μm) in direct contact with the implant at cancellous bone area (Fig. 2). For evaluation of the TBL, five equally distributed sites were chosen for each screw and the mean value of all screws was accepted as the value of the index of the section (7). Three discontinuous sections of each specimen were measured at a magnification of ×100 and the mean of the three sections was accepted as the value of the specimen for each index.

### Bone dynamic histomorphometry

For the evaluation of the bone dynamic mineralization, the following indices were measured on the surface of the bone lamellae in direct contact with the implant. The indices included mineral apposition rate (MAR, mm/day) = distance of two labeled line/number of interval days; the ratio of mineralizing surface to bone surface (MS/BS, μm/μm) = [(0.5 single labeled perimeter + double labeled perimeter) × 100]/bone perimeter; and bone formation rate per unit of bone surface [BFR/BS, μm^3^/(μm^2^day)] = MS/BS × BMR (25,26).

### Biomechanical testing

Measurement of the removal torque of the implants was performed with a method similar to a previous report (2). Briefly, the specimens were fixed in 10% neutral buffered formalin and embedded in a quadrate metal box with dental plaster. The testing equipment included a force measurementor (DZE-5, Asida, Zhengye Electronics, Dongguan, China) for recording the peak force value in newtons (N) required to loosen the implant, and a custom-made wrench that connected the implant with the force measurementor at the other. The implant used was custom-designed, with a square cap to hold the wrench. The removal torque was calculated as follows: Peak force value × distance between the force point and the center of the implant.

### Statistical analysis

The data are expressed as the means ± standard deviation, and statistical analyses were performed using SPSS software, version 12.0 (SPSS, Inc., Chicago, IL, USA). A one-way analysis of variance was conducted to assess the differences of all the quantitative indices among the three groups, and a Dunnett’s T3 test was applied for multiple comparisons. P<0.05 was considered to indicate a statistically significant difference.

## Results

One rat in the OVX group experienced a minor skin infection 5 days post-implantation, and was healed by local and systemic administration of antibiotics. All other animals tolerated the surgeries and healed without any complications, for the duration of the experiment.

### BW of rats, BMD and histological changes of the femur

The alterations in BW and BMD are presented in Table I. At the beginning of the experiment, no significant difference was identified between the BWs of the rats in the 3 groups (P=0.74, Table I), whilst significant differences were observed between the three groups at the end of the experiment. The BW of the OVX group was significantly higher than that of the sham group (P<0.01). The rats in the OVX-V group presented a significantly reduced BW as compared with that of the OVX group (P=0.021), but it remained significantly higher than the sham control group (P<0.01) and did not return to normal levels.

The femur BMDs of the three groups were similar at the beginning of the experiment (P=0.69), whilst significant differences were observed between the three groups at the end of the experiment. The BMD was reduced in the OVX group as compared with the sham group (P<0.01). The BMD of the OVX-V group was significantly higher than that of the OVX group (P=0.031), but also significantly lower than that of the sham group (P<0.05).

**Figure 3:**
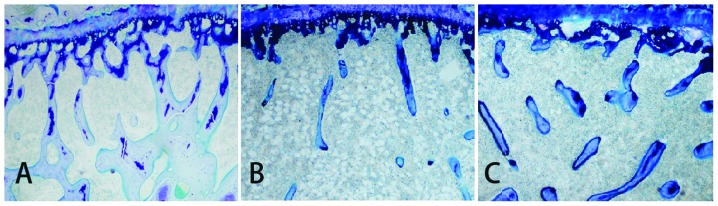
Histological examination of the distal metaphyses of the femurs using Toluidine blue in (A) sham, (B) OVX and (C) OVX-V groups at four weeks following vibration. The trabeculae near the proximal metaphysis were sparse and discontinuous in the OVX group. This condition was ameliorated in the OVX-V group, although not to the level of the sham group. Magnification, ×100. Sham, sham operation; OVX, bilateral ovariectomy; OVX-V, bilateral ovariectomy with vibration treatment.

The alterations to the femurs were also confirmed by histological examination (Fig. 3). At the end of the experiment, the trabeculae near the metaphyses of the femurs in the OVX group were sparse, thin and discontinuous, while they were thick and continuous in the sham animals. Vibration treatment markedly increased the number of the trabeculae in the OVX-V group, although they did not reach the normal level of the sham group.

### Histological and bone static histomorphometry

**Figure 4:**
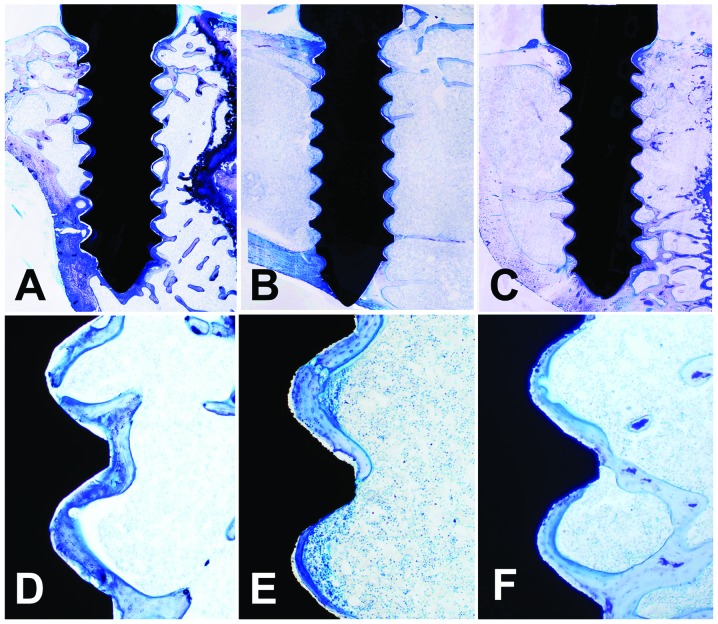
Histological images of the proximal tibiae with implants using Toluidine blue stain. (A) sham, (B) OVX and (C) OVX-V groups at four weeks following vibration (magnification, ×40) and (D) sham, (E) OVX and (F) OVX-V groups at 10 weeks (magnification, ×200). Differences were observed in the trabecular structure and implant-bone contact around the implants between the three groups. The OVX group presented the poorest implant-bone integration and the least calcified trabecular structure, while vibration treatment markedly increased the presence of implant-bone integration and calcified trabecular structure in the OVX-V group, as compared with the OVX group. Sham, sham operation; OVX, bilateral ovariectomy; OVX-V, bilateral ovariectomy with vibration treatment.

Histological images obtained from undecalcified sections are presented in Fig. 4. In the sham group, the bone lamellae in direct contact with the implant were thick and continuous, and more trabecular bone was observed around the implant. However, the bone lamellae in the OVX group were thin and discontinuous, and less trabeculae were observed. The appearances of the bone lamellae and trabeculae in the OVX-V group were much better than those in the OVX group, although the appearance was still not returned to the ‘normal’ appearance of the sham group.

Histomorphometric indices, including BIC (%), BF (%) and TBL are presented in Table II. The highest BIC, BF and TBL were identified in the sham group, while the OVX group exhibited the lowest measurements (P<0.01 as compared with the sham group). Vibration intervention significantly increased the BF and TBL in the OVX-V group as compared with the OVX group (P<0.05 and P<0.01, respectively) but all indices remained significantly lower than the sham group.

### Bone dynamic mineralization evaluation

**Figure 5:**
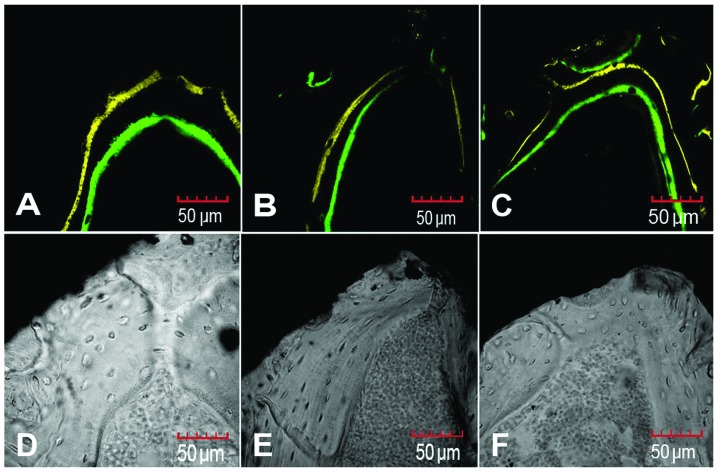
Observation of bone lamellae in direct contact with implant by fluorescence analysis. Fluorescent imaging of the (A) sham, (B) OVX and (C) OVX-V groups. Normal microscopy imaging without fluorescence in the (D) sham, (E) OVX and (F) OVX-V groups. A significant difference in fluorescence intensity, thickness and width of the double-labeled fluorescent lines was observed between the three groups. The OVX group exhibited the poorest fluorescent labeling, in which the fluorescence intensity was weak, fluorescent lines were thin and discontinuous and the distance of the double-labeled line was narrow. The vibration was markedly increased in the fluorescent labeling of the interface bone lamellae in the OVX-V group. The intensity of the fluorescence was stronger in the OVX-V group as compared with the OVX group. Magnification, ×600. Sham, sham operation; OVX, bilateral ovariectomy; OVX-V, bilateral ovariectomy with vibration treatment.

The results of LSCM fluorescence imaging of the bone tissues around the implants are presented in Fig. 5. The sham group displayed strong fluorescence intensity, thick and continuous fluorescent labeled lines and a wide distance between the two fluorescent lines. In the OVX group, the fluorescent intensity was weak, the fluorescent labeled lines were thin and discontinuous, and the distance between the two fluorescent lines was narrow. The appearance of the fluorescent labeling in the OVX-V group was markedly strengthened by vibration intervention, and appeared stronger than the fluorescence in the OVX group, although it was still not as strong as in the sham group.

The above changes were also supported by quantitative analysis (Table III). The highest bone dynamic indices, including the MAR, the MS/BS and BFR/BS were identified in the sham group, and these were significantly reduced in the OVX group (P<0.01). In the OVX-V group, MS/BS (P<0.05), MAR and BFR/BS (P<0.01) were significantly greater than in the OVX group, but significantly lower than the indices of the sham group.

### Biomechanical testing

**Figure 6:**
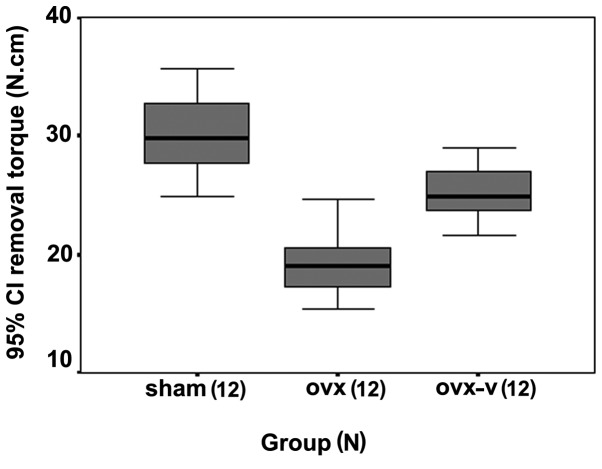
Comparison of the removal torque (N.cm) between the three groups. One-way analysis of variance indicated a P<0.01 between the three groups, and post-hoc tests indicated a P<0.01 between any two groups. Sham, sham operation; OVX, bilateral ovariectomy; OVX-V, bilateral ovariectomy with vibration treatment.

The removal torques of the titanium implants in the sham, OVX and OVX-V groups were 30.11±3.37, 19.12±2.52 and 25.30±2.17 N.cm, respectively (Fig. 6). The removal torque in the OVX-V group was significantly increased by LMHF vibration as compared with the force required for the OVX group (P<0.01), although it remained lower than that in sham-operated animals (P<0.01).

## Discussion

Osseointegrated dental implants have been widely accepted as a promising treatment modality for the rehabilitation of partially or completely edentulous patients (27,28). However, the long-term success of dental implants is determined by the quantity and quality of the host bone, which may be compromised by various systemic conditions, including postmenopausal osteoporosis (1,2,29). Currently, early and immediate implant loading have become accepted protocols for these clinical situations (30,31), but these pose great challenges to early bone healing and implant osseointegration, particularly in cases of compromised bone condition (18). This provides the motivation to explore methods that accelerate the osseointegration process in such conditions, to allow earlier functioning of dental implants.

LMHF loading by WBV, as a non-pharmacological intervention and special model of mechanical loading, has been demonstrated to have the ability to improve bone quality (15,32), strengthen bone biomechanical properties (16) and accelerate bone healing in osteoporotic bones (14). A number of studies have further demonstrated that LMHF loading produced a positive effect in peri-implant bone healing in normal animals, and the effects can be observed within one week following loading (17,18). Based on the results of the aforementioned studies, it is reasonable to speculate that LMHF vibration may exert similar bone-stimulating effects on peri-implant bone healing and implant osseointegration in osteoporotic bones.

This speculation was confirmed in part in a study by Chen *et al* (20), in which LMHF loading by WBV significantly increased bone-to-implant contact, the peri-implant bone fraction and implant mechanical properties in ovariectomized rats. The LMHF loading lasted for 8 weeks, and the effect of LMHF in the earlier stages with a shorter loading duration was not explored. Although similar anabolic bone responses, based on experience, may be expected with a 4-week loading duration, direct evidence is required to verify this theory. In another study by Akca *et al* (19), LMHF loading with a shorter duration (2 weeks) was applied and micro-computed tomography (CT) examination revealed significant enhancement of the bone volume around titanium implants in osteoporotic rats. In the study by Akca *et al* (19), only microCT analysis was performed, therefore numerous other important parameters, including bone-to-implant contact, bone dynamic indices and removal torque of implants, were not assessed. Therefore the supporting data acquired remained insufficient, and further investigation was required.

In the current study, it was hypothesized that a short 4-week period of LMHF loading by WBV can partly reverse the negative effect of osteoporosis and promote early peri-implant bone formation and implant osseointegration in osteoporotic rats. This hypothesis was tested by BMD measurement, histological examination of both static and dynamic bone parameters and biomechanical testing. Following a 4-week LMHF vibration protocol, the BMD in the OVX-V group was significantly increased as compared with that of the OVX group, although it was still less than that of the sham control. Peri-implant bone healing and osseointegration were also accelerated by LMHF vibration and all bone static parameters, including BIC, BF and TBL, and dynamic parameters, including MAR, MS/BS and BFR/BS, were significantly increased. Furthermore, these results were supported by biomechanical testing, in which the remove torque of implants was measured as being significantly increased in the vibration-treated animals. Similar anabolic effects of LMHF loading on the implant osseointegration, with the short loading duration, were also obtained from previous studies (17,21), although the animals used in previous studies were not osteoporotic.

The vibration regime, including the magnitude, frequency and loading duration, have been demonstrated to be important in influencing the effects of LMHF on peri-implant bone healing (15–18,20,24,33). In the current study, the LMHF vibration regime was 45 Hz, 0.2 g, 30 min/day and the total loading cycles were 81,000 cycles/day. The loading regime was slightly modified from the studies by Chen *et al* (20)(30~35 Hz, 0.3 g, 20 min/day) and Acka *et al* (19) (5 N/50 Hz, 14 min/day). The total loading cycles used in the present study was ~2× the total number of cycles used in these two previous studies. However, the regime used in current study was within the common vibration regimes demonstrated to promote bone healing and implant osseointegration (15–18,20,24). Further research with comparable loading regimes and total loading cycles would be valuable to elucidate the role of LMHF loading via WBV in peri-implant bone healing.

Mechanical loading has long been recognized as an important effector on bone tissue, which may lead to dynamic adaptation of bone mass, shape and architecture (21,24,34). Mechanical stimuli result in bone matrix deformation and lead to extracellular fluid movement within the bone lacunae and lacunar-canalicular networks, which creates shear force on the plasma membrane of the cellular process and drag forces on the fibrils in the pericellullar matrix (24,34). Osteocytes sense these forces (strain) and convert these signals into an anabolic response in the bone tissue. According to Frost (35), mechanical strain must be >1000 μɛ to trigger bone (re)modeling. This magnitude of strain applies only to low-frequency loading. High frequency mechanical stimuli can produce much lower magnitudes of strain, usually from 1 to 10 μɛ, which can be sensed by the bone and result in a similar or improved bone anabolic response. Under high-frequency loading, there is a ‘disruption’ of the power law relation, in which low-magnitude bone strains are most efficiently amplified and sensed by the bone (24). This may be as a result of increased mechanosensitivity to high-frequency loading, and more efficient mechanotransduction at the subcellular level (21,36).

To accelerate implant osseointegration in osteoporotic bones, pharmacological and non-pharmacological interventions have been individually investigated. In the current study, the effectiveness of LMHF via WBV, as a non-pharmacological intervention, has been demonstrated, and the results are in agreement with those reported by Chen *et al* (20) and Akca *et al* (19). The effect of LMHF loading in the present study however, was limited, and the bone parameters were not restored to the normal levels observed in the control animals. Numerous pharmaceuticals, including bisphosphonates (2,8–10), estrogen (7), simvastatin (11) and PTH (12) have also been demonstrated to have powerful osteogenic potential around the titanium implant. However, their use is limited by adverse reactions, including bisphosphonate-related osteonecrosis of the jaw (37–39), estrogen replacement therapy-induced breast cancer and coronary heart disease (40). To reduce these adverse events, a reasonable resolution is the combination of pharmacological and non-pharmacological interventions (such as LMHF loading), in which the dosage of pharmaceuticals may be markedly reduced and synergetic or addictive effects may be produced (20). This is a subject for future study.

Notably, the current experiment was performed on tibiae, which share different bone microarchitecture and biological mechanical stimuli to the jaw bone. Therefore, the results and conclusions of the present study cannot be directly applied to dental implants. Furthermore, WBV may not be a good approach in the dental clinic, as the transmission of vibration signals from the foot to the jaw may not be efficient. However, a similar loading protocol could be applied to the jaw bone and special loading apparatus should be designed to transmit LMHF vibration directly to the jaw bone, in order to generate a similar beneficial effect on the osseointegration of dental implants.

In conclusion, a 4-week short duration of LMHF loading (45 Hz, 0.2 g, 30 min/day) via WBV partly reverses the negative effects of osteoporosis and promotes early peri-implant bone healing and implant osseointegration in ovariectomized rats.

## Figures and Tables

**Table I:** Analysis of the body weight of rats and the bone mineral density of the femur.

Parameters	Time	Sham	OVX	OVX-V
Body weight (g)	Pre-exp	247.50±10.76	246.33±7.59	244.77±7.03
	Post-exp	288.40±5.73	322.18±7.78^a^	314.10±5.14^a^,^c^
Bone mineral density (g/cm^2^)	Pre-exp	0.2149±0.0119	0.2182±0.0115	0.2186±0.0107
	Post-exp	0.2238±0.0204	0.1806±0.0163^a^	0.2005±0.0185^b^,^c^

a P<0.01 vs. sham group;

b P<0.05 vs. sham group;

c P<0.05 vs. OVX group; n=12.

Sham, sham operated group; OVX, bilateral ovariectomy group; OVX-V, bilateral ovariectomy with vibration treatment.

**Table II:** Histomorphometric analysis of bone indices.

Indices	Sham	OVX	OVX-V
BIC	63.64±4.74	37.66±4.63^a^	58.09±4.56^b^
BF/TV	56.74±2.81	40.39±7.08^a^	48.05±4.18^a^,^c^
TBL	65.34±6.90	47.42±7.06^a^	55.60±3.45^a^,^d^

a P<0.01 vs. sham group;

b P<0.05 vs. sham group;

c P<0.05 vs. OVX group;

d P<0.01 vs. OVX group; n=12 in each group.

Sham, sham operation; OVX, bilateral ovariectomy; OVX-V, bilateral ovariectomy with vibration treatment; TBL, thickness of bone lamellae.

**Table III:** Dynamic indexes of bone metabolism (n=12).

Indices	Sham	OVX	OVX-V
MAR	2.63±0.25	1.64±0.32^a^	2.27±0.34^b^,^d^
MS/BS	0.24±0.06	0.15±0.02^a^	0.18±0.03^b^,^c^
BFR/BS	0.61±0.12	0.25±0.08^a^	0.43±0.11^a^,^d^

a P<0.01 vs. sham group;

b P<0.05 vs. sham group;

c P<0.05 vs. OVX group;

d P<0.01 vs. OVX group; n=12 in each group.

Sham, sham operation; OVX, bilateral ovariectomy; OVX-V, bilateral ovariectomy with vibration treatment; MAR, mineral apposition rate; MS/BS, ratio of mineralising surface to bone surface; BFR/BS, bone formation rate per unit of bone surface.
